# Dopamine and Mood in Psychotic Disorders: an ^18^F-DOPA PET study

**DOI:** 10.1001/jamapsychiatry.2025.1811

**Published:** 2025-08-13

**Authors:** Sameer Jauhar, Robert A. McCutcheon, Matthew M Nour, Mattia Veronese, Maria Rogdaki, Ilaria Bonoldi, Matilda Azis, Thomas Whitehurst, Atheeshaan Arumuham, Ellis Onwordi, Federico Turkheimer, Philip McGuire, Allan H Young, Oliver D Howes

**Affiliations:** 1 Division of Psychiatry, Imperial College London, Du Cane Road, London W12 0NN, UK; 2 Department of Psychiatry, University of Oxford, Oxford, OX3 7JX; 3 Oxford Health NHS Foundation Trust, Oxford, OX3 7JX; 4 Department of Psychosis Studies, Institute of Psychiatry, Psychology and Neuroscience, King’s College, London, SE5 8AF; 5 Max Planck University College London Centre for Computational Psychiatry and Ageing Research, London, WC1B 5EH, UK; 6 Department of Information Engineering, University of Padua; 7 Centre for Neuroimaging Sciences, Institute of Psychiatry, Psychology and Neuroscience, King’s College, London; 8 Department of Child and Adolescent Psychiatry, Institute of Psychiatry, Psychology and Neuroscience, King’s College, London; 9 South London and Maudsley NHS Foundation Trust; 10 East London NHS Foundation Trust; 11 Queen Mary University of London; 12 Institute of Clinical Sciences (ICS), Faculty of Medicine, Imperial College London, Du Cane Road, London W12 0NN, UK

## Abstract

**Importance:**

There is limited neurobiological or trial evidence guiding treatment of co-morbid affective syndromes in psychotic disorders. Given use of dopamine blocking antipsychotics, understanding dopamine function, across these mood states, is warranted.

**Objectives:**

To test for differences in dopamine synthesis capacity (Ki^cer^) between affective syndromes across psychotic disorders, and association with psychotic symptom severity.

**Design, setting and participants:**

In this cross-sectional ^18^F-DOPA PET study 38 individuals with first-episode psychosis and comorbid affective syndromes (MDE: n= 25; mixed/mania: n= 13) and 38 matched controls were recruited from early intervention services in inner-city London. Data were collected from March 2013 to February 2022.

**Main outcome and measures:**

Striatal Ki^cer^, Positive and Negative Syndrome Scale, Hamilton Depression Rating Scale, Montgomery Asperg Depression Rating Scale, Young Mania Rating Scale.

**Results:**

Mean (SD) age was 27.15 (8.89); MDE: 30.7 (12.83), mixed/mania: 23.7 (3.12), controls 25.99 (6.01). Sex distribution did not differ (male: depression 52%, mixed/mania 61.5%, controls 65.8%; p = .56). Ki^cer^ (controlling for age and sex) was significant across groups in whole striatum (F (2, 73) = 4.04, p=.02, R^2^ =0.13). People with psychosis and MDE had lower Ki^cer^ compared to those with psychosis and mixed/mania (β =0.014, SE=0.001, p=0.02), largest difference observed in limbic striatum (Cohen’s d=1.57, p<.001.) In the overall psychosis sample, higher striatal Ki^cer^ was associated with greater positive psychotic symptoms (R^2^ = .13, β = 0.011, SE = 0.001, p= .03), notably in associative striatum (R^2^= .15, p = .02). No significant association was found in the limbic striatum (p = .19).

**Conclusion and relevance:**

Dopamine synthesis capacity is lower in psychosis and co-morbid MDE than mixed/mania. Trans-diagnostically, greater positive psychotic symptoms are associated with higher dopamine synthesis capacity in associative, though not limbic striatum. This sub-region dopamine dysregulation has relevance for dopamine modulating therapeutic agents and drug discovery.

## Background

Mood episodes are common in psychotic disorders, major depressive disorder/episode (MDD/MDE) occurring in a third of people at clinical high-risk ^1^, MDE in up to half of people with first episode psychosis^2^, and in a quarter of people with schizophrenia^3^.

Psychotic symptoms occur in 10% of people with MDD^4^ and 50% in those with Bipolar Disorder (BD), moreso mania than depression (57% vs 13%)^5^. Whilst dopamine has a role in the pathoaetiology of psychosis, notably positive symptoms of schizophrenia^6^, its role in mood disorders is unclear. D_2-_blocking antipsychotics are effective in acute and maintenance phases of mania and psychotic disorders; this is uncertain in psychotic major depression.

Molecular imaging of the dopamine system is understudied in affective disorders, compared to schizophrenia, where associative striatum is implicated^6^. In MDD, meta-analysis suggests reduced dopamine transporters in limbic striatum^7^, consistent with compensatory downregulation, due to lowered dopamine signalling. In BD, one suggested elevated dopamine synthesis capacity (Ki^cer)^) in psychotic BD^8^, another no elevation in non-psychotic mania^9^. The former found association between Ki^cer^ and positive psychotic symptoms transdiagnosttically across BD and schizophrenia.

No studies have examined Ki^cer^ across depressive episodes and psychotic disorders or in comparison to mixed/mania psychosis.

### Aims and objectives

We examined Ki^cer^ in psychosis with MDE or mixed/mania, and whether positive psychotic symptoms were associated with Ki^cer^ across mood states. We hypothesized that, in psychosis, MDE would be associated with lower Ki^cer^ than mixed/mania, specifically in limbic striatum. The secondary hypothesis was that Ki^cer^ would correlate with positive psychotic symptoms, across affective disorders, specifically associative striatum. Our exploratory hypothesis was that Ki^cer^ would be inversely correlated with depression symptoms.

## Methods

Ethical permission was obtained from East of England-Cambridge East and local Research Ethics Committee, and Administration of Radioactive Substances Advisory Committee.

Written informed consent was obtained from all participants.

Details of all three samples are in Supplementary Material.

### Inclusion criteria

(1) First episode psychosis and psychotic symptoms (including Brief limited psychotic symptoms), defined previously^8^.
(2) Current mood:
  i) MDE, on interview (Mini-International Neuropsychiatric Interview), confirmed using Hamilton Depression Rating Scale (HDRS) cut-off ≥ 14 (moderate depression)/**Montgomery–Åsberg** Depression Rating Scale (MADRS)≥ 20 (moderate depression). OR
  ii) Mixed/mania, Young Mania Rating Scale (YMRS) ≥ 4^12^ and depression, as above.

### Exclusion criteria

Head trauma, dependence on illicit substances, opiates/alcohol misuse, significant medical co-morbidity, contra-indications to PET, taking mood stabilizers.

### Clinical measures

PANSS, HDRS/MADRS, YMRS.

### ^18^F-DOPA PET imaging

#### Data acquisition and analysis

See Supplementary Material

### Statistical Analysis

SPSS Version 29, significance set as p<0.05 (two-tailed).

To test our primary hypothesis we used linear regression, striatal Ki^cer^ as dependent variable, MDE (versus mixed/mania) as predictors, age and sex as covariates, due to effects on Ki^cer^ and unbalanced ages in our sample. We repeated analysis in striatal functional subdivisions. For the secondary hypothesis we performed linear regressions, positive psychotic symptoms as dependent and Ki^cer^ as predictor.

Exploratory linear regressions examined associations between striatal subdivisions and depression severity. Outliers were identified using Cook’s distance (4/n)^8^. As two depression scales were used (HAM-D, MADRS), acknowledging heterogeneity, using a three factor validated composite measure (observed mood, cognitive, neurovegetative symptoms).

## Results

76 people were included (25 MDE, 13 mixed/mania, 38 propensity score-matched controls). Patient populations have been reported previously; 16 in^11^, and differing 16 in^12^, 13 in ^8^, data on 7 not previously reported.

Diagnoses were MDD (n=15), Schizophrenia (n=7), BD (n=15), MDD, co-morbid PTSD (n=1). 2 of the MDE group had BD. Diagnoses were stable at 5 years for studies 1 and 2; 3 years for study 3.)

### Ki^cer^ across groups

In whole striatum, there was significant effect of diagnostic group, adjusting for age and sex (F (2,71) = 4.04, p = .02, partial η^2^ = 0.10). The overall model was significant (F (4,71) = 2.66, p = .04, R^2^ = 0.13).

Findings remained significant, controlling for chlorpromazine dose years, smoking and ethnicity (p=.04). Analysis of subdivisions revealed greatest difference between MDE and mixed/mania groups in limbic striatum (β = 0.015, SE = 0.001, p = .001). Pairwise comparisons showed significant difference between MDE (though not mixed/mania) and controls (p=.004, p=.19).

### Relationship between Ki^cer^ and positive psychotic symptoms

Two outliers were identified.

Striatal Ki^cer^ was associated with positive symptoms (R^2^ = .13, β = 0.000066, SE = 0.000030, p = .03), higher Ki^cer^ related to greater positive symptom severity. This remained significant after controlling for mood group (depression vs mixed/mania) (R^2^ = .26, β = 0.000057, SE = 0.000028, p = .049). Regionally, this was strongest in associative (R^2^ = .15, β = 0.000076, SE x= 0.000031, p = .02), sensorimotor (R^2^ = .11, β = 0.000068, SE = 0.000034, p = .05), though non-significant in limbic striatum (R^2^ = .05, β = 0.000036, SE = 0.000027, p = .19).

### Relationship between Ki^cer^ and depression symptom clusters

Linear regression revealed no relationship between Ki^cer^ and observed mood/cognition (p>0.05), significant association between neurovegetative factor, limbic (R^2^ = .18, β = 0.002, p = .03; standardized β = 0.434) and sensorimotor striatum (R^2^ = .17, β = 0.00262, SE = 0.00119, p = .04).

No associations were observed with other regions (p>.05).

## Discussion

We found lower Ki^cer^ in psychosis with MDE, compared to mixed/mania, notably in limbic striatum, consistent with imaging of dopamine transporters in depression^7^ and fMRI model of mania.^13^. Conversely, association between positive symptoms and Ki^cer^ was pronounced in *associative* striatum, consistent with meta-analysis in schizophrenia versus controls^6^.The relationship between limbic/sensorimotor Ki^cer^ and neurovegetative factor may be explained by this including sleep and appetite. Our findings suggest compensatory mechanisms, decreased D_2/3_ receptor availability observed in sleep deprivation, consistent with increased endogenous dopamine^14^.

### Strengths and limitations

Most of our first episode sample were free of psychotropic medicines; controlling for antipsychotics did not alter results. The same scanner and protocols were used.

Limitations included lack of non-affective psychosis group, variable depression measures; validated criteria were used to mitigate this. Combining mixed states/mania is justified, with FDA-approved antipsychotics for mixed states/mania.^7^ We acknowledge low YMRS scores., and factor constructs for MDE.

Diagnostic constructs can change, though did not occur here. Finally, we included individuals from a prior study examining association between Ki^cer^ and positive symptoms; controlling for mood (depression, mixed/mania) increased magnitude of association.

### Theoretical and therapeutic implications

Affective psychoses involve disruption of systems underlying emotional regulation and motivation, while non-affective psychoses are linked to executive dysfunction. These symptomatic differences map onto distinct patterns of dopaminergic dysregulation: the limbic striatum, involved in reward processing, connects to medial prefrontal and limbic cortical regions; in contrast, the associative striatum connects to dorsolateral prefrontal and parietal cortices supporting cognitive control. These differences suggest nuanced therapeutic targets across psychotic disorders. This aligns with cariprazine’s effectiveness in unipolar and bipolar depression. with higher limbic D_3_ affinity (potentially agonism). Moreover, low-dose amisulpride is effective in depression, acting on the pre-synaptic auto-receptor, with limbic selectivity. Lumateperone (pre-synaptic D_2_R partial agonist and post-synaptic D_2_R antagonist) has mesolimbic and mesocortical selectivity, and effectiveness in bipolar depression.

### Conclusions and future directions

We found lowered Ki^cer^ in psychosis and depression, compared to mixed/mania, especially in limbic striatum. Conversely, association between positive psychotic symptoms and Ki^cer^ in affective disorders was notable in associative, not limbic striatum. Understanding these regional differences may refine treatments across psychotic disorders.

## Supplementary Material

Supplementary Material

## Figures and Tables

**Figure 1:**
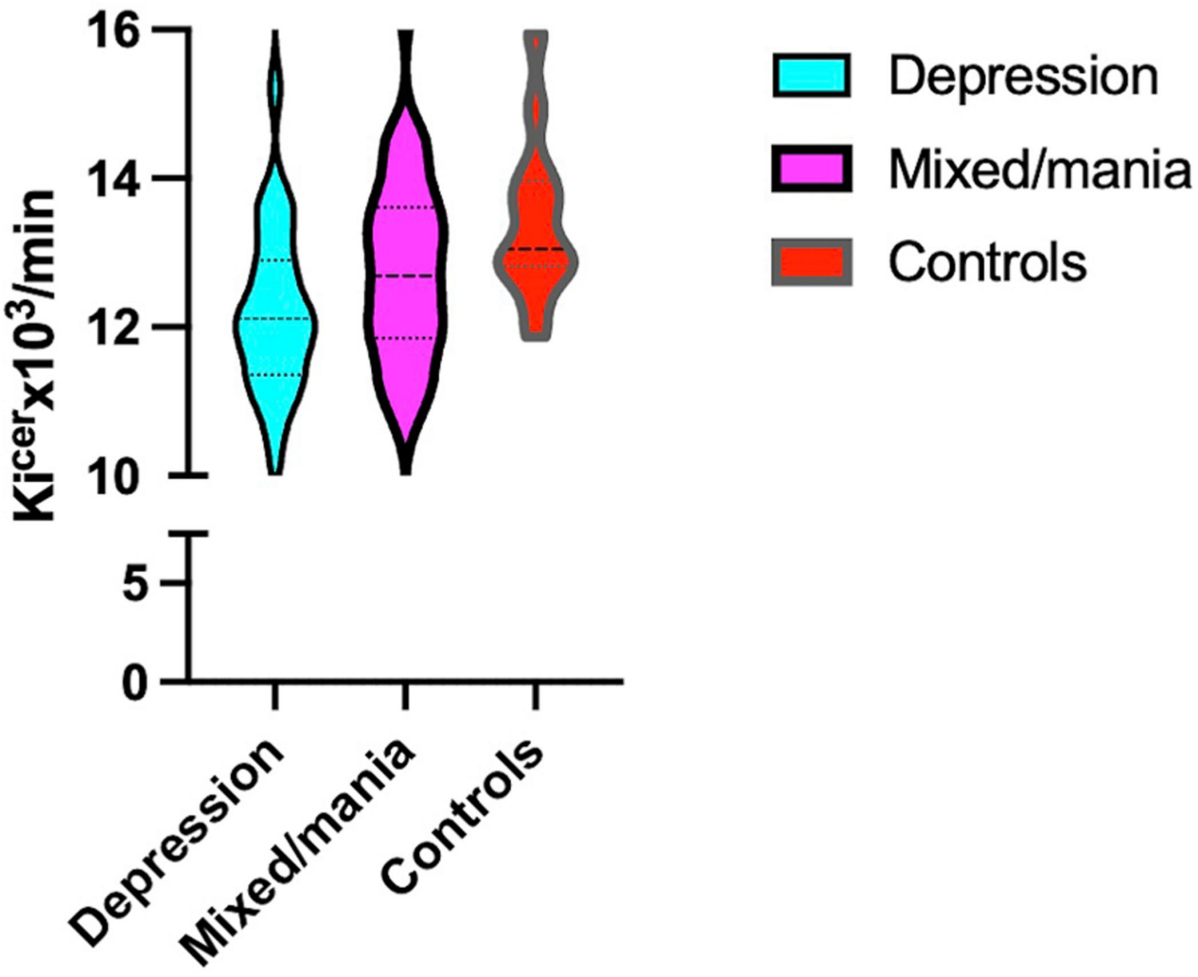
Striatal Dopamine Synthesis Capacity Across Diagnostic Groups Violin plot showing the distribution of dopamine synthesis capacity (Ki^cer^, expressed as ×10^3^/min) in whole striatum across diagnostic groups: individuals with psychosis and current major depressive episode (*n* = 25), mixed/mania (*n* = 13), and healthy controls (*n* = 38). The width of each violin reflects the kernel density estimate of the data distribution. Horizontal lines indicate the median and interquartile range. A significant group effect was observed (F(2, 71) = 4.04, *p* = .02), with post hoc comparisons showing significantly lower Ki^cer^ in the depression group versus the mixed/mania group, and versus controls

**Figure 2:**
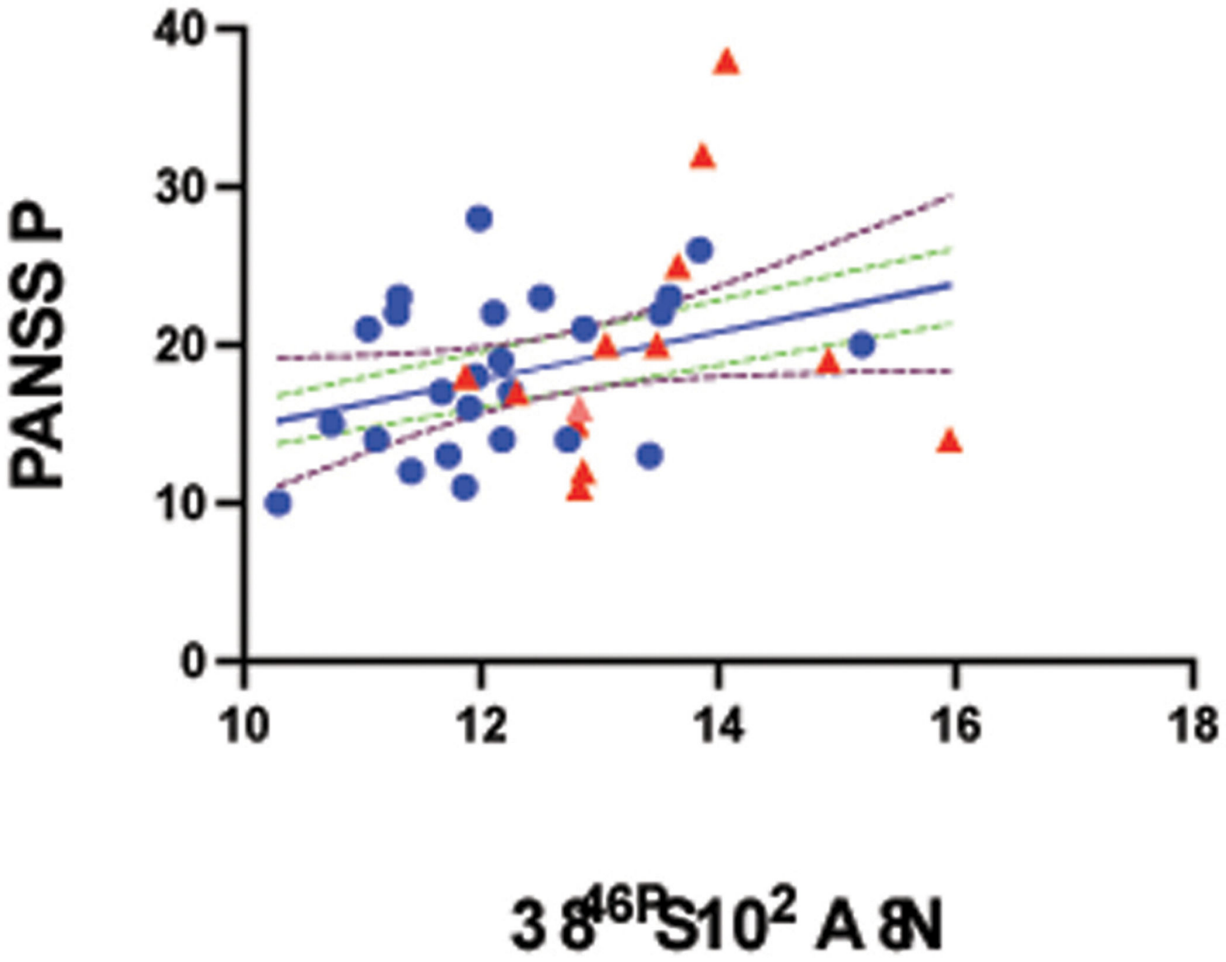
Association Between Whole Striatal Dopamine Synthesis Capacity and PANSS Positive Psychotic Symptom scores Scatterplot showing the relationship between dopamine synthesis capacity in the whole striatum (Ki^cer^ ×10^3^/min) and PANSS positive symptom scores in individuals with psychosis and a current affective episode (n = 38). Individuals with major depressive episode are shown as blue circles; those with mixed/mania as red triangles. A significant positive association was observed (R^2^ = .13, β = 0.000066, SE = 0.000030, p = .03), indicating that higher Ki^cer^ was associated with greater positive symptom severity. Dotted lines represent the 95% confidence interval of the regression line.

**Table 1:** Demographic. clinical and imaging details

Measure	Depression (N=25)	Mixed/mania (N=13)	Controls (N=38)	p value
Age, years, Mean (SD)	30.71 (12.83)	23.69 (3.12)	25.99 (6.01)	0.03
Sex (% Male)	52	61.5	65.8	0.56
Ethnicity % White/Black/Other	60/20/20	46/39/15	37/8/55	0.01
Antipsychotic status	48% naive 20% free 4% min treated 28% current	61.5% naive 30.8% free 7.7% current		
Cigarette smoking (% smokers)	32%	76.9%	31%	<0.001
Radioactivity injected (MBq), Mean (SD)	156.97 (16.09)	146.84 (18.78)	158.01 (14.5)	0.09
Specific activity (GBq/μmol), Mean (SD)	0.03 (0.01)	0.03 (0.01)	0.03 (0.02)	0.93
PANSS Positive, Mean (SD)	18.4 (4.76)	19.77 (7.79)		0.49
PANSS Negative, Mean (SD)	15.44 (5.34)	13.54 (6.46)		0.34
PANSS General, Mean (SD)	37.84 (9.24)	33.77 (9.57)		0.21
PANSS Total, Mean (SD)	71.68 (16.66)	67.08 (19.46)		0.45
				
Striatal Region	Mean (SEM) Ki^cer^ ×10^3^/min	Mean (SEM) Ki^cer^ ×10^3^/min	Mean (SEM) Ki^cer ^×10^3^/min	Effect size (Cohen’s d, 95% CI) / p
Whole striatum	12.23 (0.22)	13.42 (0.31)	12.79 (0.20)	1.08 (0.36–1.79) / 0.04
Associative striatum	12.23 (0.24)	13.42 (0.34)	12.80 (0.19)	0.99 (0.27–1.69) / 0.06
Limbic striatum	12.00 (0.18)	13.32 (0.21)	12.74 (0.19)	1.57 (0.80–2.33) / 0.01
Sensorimotor striatum	12.33 (0.25)	13.37 (0.35)	12.68 (0.25)	0.80 (0.10–1.49) / 0.06

## Key points

### Question

Is dopamine synthesis capacity altered in the presence of mood symptoms in individuals with a psychotic disorder, and is there an association with positive psychotic symptoms across affective syndromes?

### Findings

In individuals with psychosis, decreased striatal dopamine synthesis capacity was observed in depression episode, compared to mixed/mania syndromes. This was most notable in limbic striatum. Across affective syndromes, a relationship was observed between positive psychotic symptoms and dopamine synthesis capacity, most pronounced in associative striatum.

### Meaning

Dopamine function differs across psychotic disorders when different mood states are present, particularly limbic striatum. Trans-diagnostically, positive psychotic symptoms are related to dopamine synthesis capacity in associative striatum. These findings have relevance for understanding the aetiology of psychosis and therapeutic agents for people with co-morbid affective syndromes and psychotic disorders.
