# Supplementary material for: Dopamine and Mood in Psychotic Disorders: an 18F-DOPA PET study
Source: JAMA Psychiatry. Author manuscript; Available in PMC 2025 Sep 19. (PMC12351467; doi:10.1001/jamapsychiatry.2025.1811)
Supplement: Supplementary Material [file EMS207969-supplement-Supplementary_Material.docx]

**Supplementary Material Dopamine and Mood in Psychotic Disorders: an ^18^F-DOPA PET Study**

**eMethods 1. Study Inclusion**

**eMethods 2. Depression factor scoring**

**eFigure 1. Relationship between sensorimotor Ki and neurovegetative factor score**

**eFigure 2. Relationship between limbic Ki and neurovegetative factor score**

**eMethods 1. Study Inclusion**

The sample consisted of people from three cohorts, two of which have been published previously^1,2^. The third study was a study in people with psychotic major depression (PMD), defined using the Mini International Neuropsychiatric Interview (MINI) as Major Depressive Episode (MDE) with psychotic features, in people presenting for the first time to services.

**eMethods 2. Depression factor scoring**

Depression factors were taken from Uher et al^3^, with items that loaded>0.5.

Scores from scales were entered as percentage of total score.

**Observed mood**

MADRS (items)1,2,3,7

HAM-D (items) 1,7,9,10,11.13

**Cognitive**

MADRS 9, 10

HAM-D 2, 3

**Neurovegetative**

MADRS 4, 5

HAM-D 4,5,6,12,14,16

N.b. sexual function item taken out, due to relationship to antipsychotic use

**eMthods 3. PET data acquisition**

All participants were asked not to eat or drink (except water), and refrain from alcohol for 12 hours prior to scan. Cigarette smokers were not permitted to smoke in the four hours preceding the scan. The four hour cut-off for last cigarette smoked was based on evidence that nicotine’s occupancy of the acetylcholine nicotinic receptor does not change appreciably between 2-5 hours after last administration of nicotine^4^.

Imaging data for all studies were obtained on the same Siemens Biograph 6 HiRez PET scanner (Siemens, Erlangen, Germany), in three-dimensional mode. Participants received 400 mg entacapone, a peripheral catechol-*o*-methyl-transferase inhibitor, and 150 mg carbidopa, a peripheral aromatic acid decarboxylase inhibitor one hour prior to scanning, to prevent formation of radiolabeled metabolites that may cross the blood–brain barrier ^5^.

Participants were positioned in the scanner with the orbitomeatal line parallel to the transaxial plane of the tomograph. Head position was marked, monitored and movement minimized, using a head strap. After acquiring a CT scan for attenuation correction, ^18^F-DOPA was administered by bolus intravenous injection 30 seconds after start of PET imaging. PET data were acquired in 32 frames of increasing duration over the 95-minute scan (frame intervals: 8x15 seconds, 3x60 seconds, 5x120 seconds, 16x300 seconds).

**eMethods 3. PET Data analysis**

The region-of-interest (ROI) analysis was conducted blind to clinical status. Our primary endpoint was Ki^cer^ (K_i_ in previous publications^6^) for the whole striatum. Secondary analyses were conducted with striatal subdivisions (associative, limbic and sensorimotor), defined using the atlas by Martinez et al^7^.

Correction for head movement during scans employed a mutual information algorithm ^8^. SPM 8 was used to automatically normalize a tracer-specific ^18^F-DOPA template^9^, together with the striatal brain atlas, defined by Martinez et al^7^ and Hammersmith brain atlas. The Hammersmith brain atlas was used to identify extra-striatal regions and the reference region (cerebellum). The ROI atlas was transformed into the subject’s PET space, using the tracer-specific template without use of a co-registered MRI. This method has demonstrated good reliability in a previous test-retest study ^11^. Ki^cer^ was calculated using the Patlak-Gjedde graphical approach, adapted for a reference tissue input function ^12^. Further details of our image analysis approach are given in prior publications^6,13^.

**Supplementary eTable 1**

**eTable 1. Psychotropic medication at time of scan**

| **Depression sample (N=25)**  **All n=1** | **Mixed/mania sample (N=13)**  **All n=1** |
| --- | --- |
| Amisulpride 300mg | Amisulpride 300mg |
| Amisulpride 600mg, amitriptyline 100mg |  |
| Aripiprazole 15mg, sertraline 50mg |  |
| Aripiprazole depot 400mg monthly, lofepramine 140mg |  |
| Quetiapine 25mg |  |
| Quetiapine 300mg, mirtazapine 15mg |  |
| Quetiapine 100mg, sertraline 100mg |  |
| Mirtazapine 15mg |  |

**Psychotropic medication and doses (mg/day) for individual participants in the depression (N=25) and mixed/mania (N=13) groups. Each row reflects a single participant.**

**Supplementary eTable 2. Striatal Ki^cer^ for diagnostic groups**

| **Striatal region** | **Mixed (n=7)**  **Mean (s.e.m.)** | **Mania (n=6)**  **Mean (s.e.m.)** | **Depression (n=25)**  **Mean (s.e.m.)** |
| --- | --- | --- | --- |
| **Whole striatum** | **13.51 (0.33)** | **13.32 (0.57)** | **12.23 (0.22)** |
| **Associative striatum** | **13.5 (0.34)** | **13.32 (0.66)** | **12.23 (0.24)** |
| **Limbic Striatum** | **13.23 (0.21)** | **13.39 (0.41)** | **12.00 (0.18)** |
| **Sensorimotor striatum** | **13.64 (0.43)** | **13.0 (0.56)** | **12.33 (0.25)** |

**eResults 1. Relationship between dopamine synthesis capacity and positive psychotic symptoms**

Including outliers, the following were observed:

For whole striatum (R^2^=0.09, co-efficient=0.011, SE=0.001, p=.07).

For associative striatum (R^2^=0.1, co-efficient=0.011, SE=0.001, p=.05).

For limbic striatum (R^2^=0.03, co-efficient=0.012, SE=0.001, p=.28).

For sensorimotor striatum (R^2^=0.03, co-efficient=0.01, SE=0.001, p=0.06).

**eResults 2. Relationship between Ki^cer^ and depression symptom clusters**

Sensorimotor striatum

Linear regression revealed a significant positive association between dopamine synthesis capacity in the sensorimotor striatum and neurovegetative symptom severity. The model explained 17% of the variance (R² = .17), with an unstandardized coefficient (β = 0.00262, SE = 0.00119, p = .04; standardized β = 0.417).

Limbic striatum

Linear regression revealed a significant positive association between dopamine synthesis capacity in the limbic striatum (Ki^cer^) and neurovegetative symptom severity. The model explained 13.6% of the variance (R² = .14), with an unstandardized coefficient (β = 0.002, SE = 0.001, p = .03; standardized β = 0.434).

After exclusion of an outlier identified using Cook’s distance (> 4/n), the association remained statistically significant (R² = .14, adjusted R² = .11, standardized β = 0.369, p = .03).
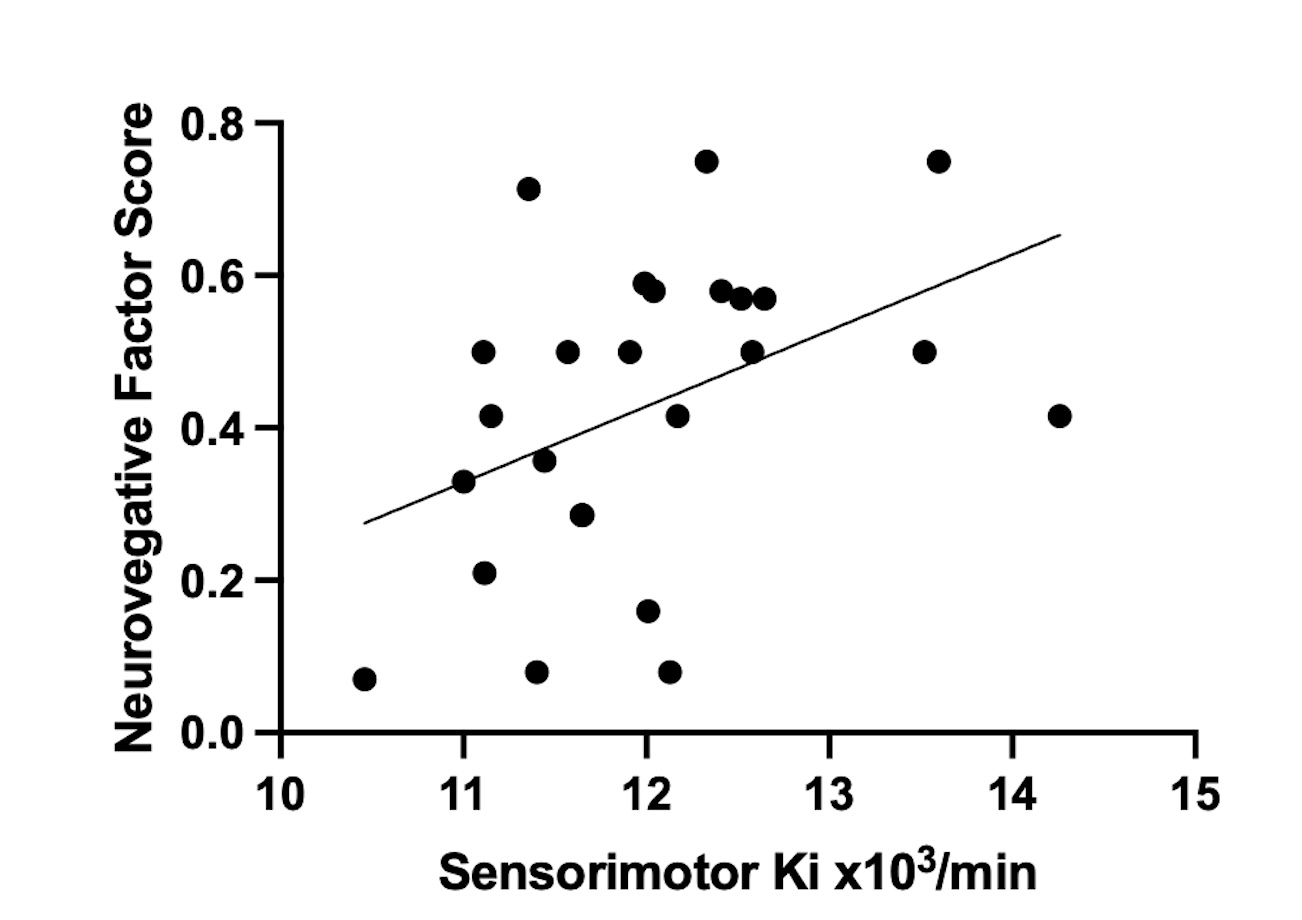


**eFigure 1.**

**Association Between Sensorimotor Striatal Dopamine Synthesis Capacity and Neurovegetative Factor Score in Depression**

**Scatterplot showing the association between dopamine synthesis capacity in the sensorimotor striatum (Ki^cer^, ×10³/min) and neurovegetative factor score in individuals with depression (n = 25). After exclusion of an outlier identified using Cook’s distance (> 4/n), the association remained statistically significant (R² = .17, adjusted R² = .14, standardized β = 0.417, p = .04).**

**
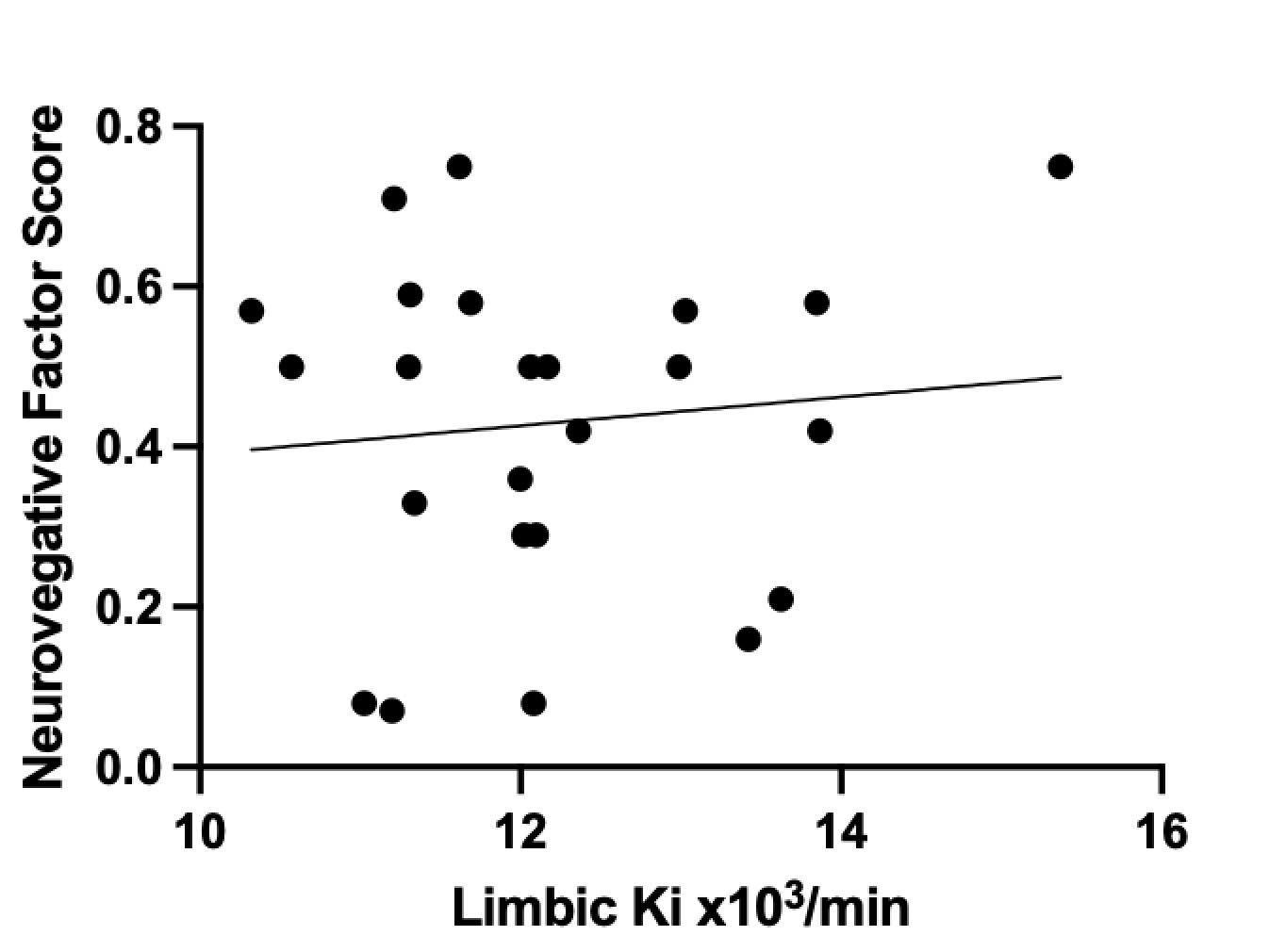
**

**eFigure 2. Association Between Limbic Striatal Dopamine Synthesis Capacity and Neurovegetative Factor Score in Depression**

**Scatterplot showing the association between dopamine synthesis capacity in the limbic striatum (Kicer, ×10³/min) and neurovegetative factor scores in individuals with depression (*n* = 25). A significant association was observed (R² = .18, β = 0.002, p = .03; standardized β = 0.434). After exclusion of an outlier identified using Cook’s distance (> 4/n), the association remained statistically significant (R² = .14, adjusted R² = .11, standardized β = 0.369, p = .03).**

**References**

1. Jauhar S, Veronese M, Nour MM, et al. Determinants of treatment response in first-episode psychosis: an 18 F-DOPA PET study. *Molecular Psychiatry*. 2019;24(10):1502-1512. doi:10.1038/s41380-018-0042-4

2. Howes OD, Bonoldi I, McCutcheon RA, et al. Glutamatergic and dopaminergic function and the relationship to outcome in people at clinical high risk of psychosis: a multi-modal PET-magnetic resonance brain imaging study. *Neuropsychopharmacol*. 2020;45(4):641-648. doi:10.1038/s41386-019-0541-2

3. Uher R, Farmer A, Maier W, et al. Measuring depression: comparison and integration of three scales in the GENDEP study. *Psychological Medicine*. 2008;38(2):289-300. doi:10.1017/S0033291707001730

4. Esterlis I, Mitsis EM, Batis JC, et al. Brain β2*-nicotinic acetylcholine receptor occupancy after use of a nicotine inhaler. *Int J Neuropsychopharmacol*. 2011;14(3):389-398. doi:10.1017/S1461145710001227

5. Cumming P, Léger GC, Kuwabara H, Gjedde A. Pharmacokinetics of plasma 6-[18F]fluoro-L-3,4-dihydroxyphenylalanine ([18F]Fdopa) in humans. *J Cereb Blood Flow Metab*. 1993;13(4):668-675. doi:10.1038/jcbfm.1993.85

6. Jauhar S, Veronese M, Rogdaki M, et al. Regulation of dopaminergic function: an [18F]-DOPA PET apomorphine challenge study in humans. *Transl Psychiatry*. 2017;7(2):e1027. doi:10.1038/tp.2016.270

7. Martinez D, Narendran R, Foltin RW, et al. Amphetamine-Induced Dopamine Release: Markedly Blunted in Cocaine Dependence and Predictive of the Choice to Self-Administer Cocaine. *AJP*. 2007;164(4):622-629. doi:10.1176/ajp.2007.164.4.622

8. Turkheimer FE, Brett M, Visvikis D, Cunningham VJ. Multiresolution analysis of emission tomography images in the wavelet domain. *J Cereb Blood Flow Metab*. 1999;19(11):1189-1208. doi:10.1097/00004647-199911000-00003

9. Howes OD, Montgomery AJ, Asselin MC, et al. Elevated striatal dopamine function linked to prodromal signs of schizophrenia. *Arch Gen Psychiatry*. 2009;66(1):13-20. doi:10.1001/archgenpsychiatry.2008.514

10. Hammers A, Allom R, Koepp MJ, et al. Three-dimensional maximum probability atlas of the human brain, with particular reference to the temporal lobe. *Hum Brain Mapp*. 2003;19(4):224-247. doi:10.1002/hbm.10123

11. Egerton A, Demjaha A, McGuire P, Mehta MA, Howes OD. The test-retest reliability of 18F-DOPA PET in assessing striatal and extrastriatal presynaptic dopaminergic function. *Neuroimage*. 2010;50(2):524-531. doi:10.1016/j.neuroimage.2009.12.058

12. Patlak CS, Blasberg RG. Graphical evaluation of blood-to-brain transfer constants from multiple-time uptake data. Generalizations. *J Cereb Blood Flow Metab*. 1985;5(4):584-590. doi:10.1038/jcbfm.1985.87

13. Jauhar S, Nour MM, Veronese M, et al. A Test of the Transdiagnostic Dopamine Hypothesis of Psychosis Using Positron Emission Tomographic Imaging in Bipolar Affective Disorder and Schizophrenia. *JAMA Psychiatry*. 2017;74(12):1206-1213. doi:10.1001/jamapsychiatry.2017.2943, 10.1001/jamapsychiatry.2017.2943
